# Repeatability and reproducibility of rapid T_1_ mapping of brain tissues at 64 mT: A multicentre study

**DOI:** 10.1162/IMAG.a.916

**Published:** 2025-10-08

**Authors:** Beatrice Lena, Francesco Padormo, Rui Pedro A.G. Teixeira, Carly Bennallick, James Gholam, Ruben van den Broek, Samson Lecurieux Lafayette, Irene Vavasour, Mara Cercignani, Derek K. Jones, Shannon Kolind, Jo Hajnal, Niall Bourke, Yiming Dong, William J. Hollander, Todor Karaulanov, Sean C.L. Deoni, Steven C.R. Williams, Pia C. Sundgren, Andrew G. Webb, Emil Ljungberg

**Affiliations:** C.J. Gorter MRI Center, Leiden University Medical Center, Leiden, Netherlands; Hyperfine Inc., Guilford, CT, United States; Department of Neuroimaging, Institute of Psychiatry, Psychology & Neuroscience, King’s College London, London, United Kingdom; Cardiff University Brain Research Imaging Centre (CUBRIC), School of Psychology, Cardiff University, Cardiff, United Kingdom; Perinatal Imaging & Health, King’s College London, London, United Kingdom; Department of Radiology, University of British Columbia, Vancouver, British Columbia, Canada; Department of Medicine (Neurology), Radiology, and Physics, University of British Columbia, Vancouver, British Columbia, Canada; CaliberMRI, Boulder, CO, United States; MNCH D&T, Bill & Melinda Gates Foundation, Seattle, WA, United States; Institution of Clinical Sciences/Radiology, Lund University, Lund, Sweden; Lund Bioimaging Centre, Lund University, Lund, Sweden; Department of Medical Radiation Physics, Lund University, Lund, Sweden

**Keywords:** low-field MRI, quantitative MRI, T1 mapping, multicenter, reproducibility, repeatability

## Abstract

Very-low-field MRI (<100 mT) holds promise for Point-of-Care brain imaging applications, including stroke and multiple sclerosis, with T_1_ mapping emerging as a key biomarker for brain development and pathology. However, current low-field T_1_ mapping protocols suffer from long acquisition times and limited multi-site repeatability. This study aimed to improve T_1_ mapping at 64 mT using a clinically feasible 10-minute protocol and assess repeatability and reproducibility across sites. We present an analysis of the repeatability and reproducibility of rapid T_1_ measurements in a commercially available phantom and in 60 volunteers, scanned with a portable 64 mT MRI systems at six sites. T_1_ mapping was performed using an undersampled 3D inversion-recovery turbo spin-echo sequence with a 10.8-minute scan time, and reconstructed with a locally low-rank approach. Our results in phantom demonstrated high reproducibility in T_1_ measurements (below 3% differences from the average), with non-significant differences between sites. Longitudinal measurements demonstrated high repeatability over time both in vivo and in phantom settings in one site, with minimal variability (average Coefficient of Variation of 0.6%). Average in vivo T_1_ values for white matter and cortex were 290 ± 6 ms and 332 ± 8 ms, respectively and the values demonstrated high reproducibility, with differences of less than 4% from the average across sites. Our results demonstrate the feasibility of multi-site in vivo T_1_ mapping at 64 mT, providing normative T_1_ values at this field strength and supporting its use as a quantitative biomarker in clinical applications.

## 1 Introduction

Quantitative measurement of the longitudinal relaxation time (T_1_) is a valuable tool in both clinical and research MRI applications. T_1_ mapping has been utilized to assess myocardial fibrosis in cardiomyopathy (Taylor et al., 2016), liver fat quantification (Fellner et al., 2023), gadolinium-free assessment of kidney function (Wu et al., 2021), to study white matter maturation and myelination during neurodevelopment (Deoni et al., 2012), to monitor demyelination and remyelination in neurological conditions like multiple sclerosis (O’Muircheartaigh et al., 2019), and to quantify structural changes associated with Alzheimer’s disease and other forms of cognitive decline (Wang et al., 2022). Additionally, T_1_ serves as a biomarker for assessing water content (Fatouros et al., 1991) and tissue integrity, making it a versatile metric for investigating both normal physiology and a range of pathological conditions across various tissues.

The use of very-low-field (<100 mT) MRI shows promise for applications such as stroke diagnosis (Mazurek et al., 2021), multiple sclerosis monitoring (Arnold et al., 2022), and brain morphology analysis (Iglesias et al., 2023). Given the significant variation in T_1_ values across fields—from <1 mT to 7 T (Rooney et al., 2007) due to its dependence on the Larmor frequency—precise T_1_ mapping at low field is essential to establish normative values and to utilise T_1_ as a quantitative biomarker in health and disease.

Recent studies have demonstrated the feasibility of T_1_ mapping at very low field in both adult (Jordanova et al., 2023; O’Reilly & Webb, 2022) and neonatal populations (Padormo et al., 2023). To facilitate the clinical and research translation of this technique, two critical challenges must be addressed. First, the inherently lower signal-to-noise ratio (SNR) at low field requires longer acquisition times to achieve acceptable image quality. For clinical viability however, acquisition times must be minimized. Second, a major advantage of very-low-field systems is their portability and operation without requiring a shielded room. However, this introduces a significant challenge due to electromagnetic interference (EMI). The EMI environment is subject to variation over time and between different sites, potentially affecting image quality (Srinivas et al., 2022), and impacting quantitative T_1_ measurements.

This study investigates the repeatability and reproducibility of measurements acquired at very-low-field MRI in phantom and healthy volunteers across multiple sites with a T_1_ mapping method, with acquisition time of 10 minutes. Specifically, we aim to:

- assess the reproducibility of rapid T_1_ measurements across sites using a quantitative phantom;
- evaluate the longitudinal repeatability of the T_1_ estimates for the same subject and phantom at a single site;
- investigate test-retest repeatability of the T_1_ measurements at each site in vivo.
- investigate the reproducibility of the T_1_ measurements across sites in vivo.

## 2 Methods

### 2.1 Materials and study organisation

All data were acquired at 64 mT using the Hyperfine Inc. Swoop system, which employed an integrated EMI rejection system and a single-channel transmit/eight-channel receive head coil. The participating sites, all part of the UNITY consortium (Abate et al., 2024), were equipped with identical Hyperfine Swoop systems running software version 8.7 beta. Each site was also equipped with a quantitative phantom (CaliberMRI, Boulder, CO, model 137) used for the T_1_ measurements. The phantom included a set of 14 spheres doped with NiCl_2_ to span a wide range of T_1_ relaxation times (Stupic et al., 2021). To ensure consistent and repeatable placement within the coil, the phantom was positioned using a dedicated holder, specifically designed for this purpose, as described in Ljungberg et al. (2025).

Six sites participated in the project: Cardiff Brain Research Imaging Centre (Cardiff), United Kingdom; Leiden University Medical Center (Leiden), Netherlands; Lund University (Lund), Sweden; King’s College London Centre for Neuroimaging Sciences (London 1) and King’s College London St Thomas Hospital Perinatal Imaging and Health (London 2), United Kingdom; and University of British Columbia (Vancouver), Canada. Each site recruited and scanned 10 healthy adult volunteers (>18 years old), with the inclusion criteria of no contraindications for MRI or pre-existing neurological conditions (details can be found in Supplementary S1). Ethical approval was obtained at each site, and all subjects provided written informed consent prior to involvement in the study.

#### 2.1.1 MR acquisition

The scanning protocol consisted of a prescan calibration, localizer, three T_2_-weighted scans with anisotropic voxel dimensions (axial, coronal, and sagittal) provided by the manufacturer as part of the system’s clinical product, and multiple custom inversion-recovery T_1_ mapping sequences created using additional research tools (details in Table 1). The T_1_ mapping protocol utilised a 3D inversion-recovery turbo spin-echo (TSE) sequence with an adiabatic full passage hyperbolic secant inversion pulse and six inversion times logarithmically spaced between 50 and 999 ms. K-space encoding along the two phase encoding directions used variable density random sampling with an elliptical k-space shutter with four-times undersampling, cartesian encoding along the readout direction, and centre-out sampling within each shot. The T_1_ mapping scan time was 10.8 minutes, with 01:43 minutes per inversion time. This protocol was previously optimized for a range of T_1_-values between 100 and 1000 ms (Lena et al., 2024).

**Table 1.** Summary of the MR scan parameters for each sequence.

**Sequence**	**T_2_-weighted scans**	**IR T_1_-weighted scans**
Echo time (ms)	170	5
Repetition time (ms)	1600	1500
Echo train length	60	32
Echo spacing (ms)	6	5
Inversion delay (ms)	-	50/91/166/302/549/999
Field of view [RL x AP x FH] (mm^3^)	180 × 220 × 200	180 × 220 × 200
Matrix	120 × 146 × 40	72 × 88 × 58
Voxel size (mm^3^)	1.5 × 1.5 × 5	2.5 × 2.5 × 3.5
Orientation	Axial/Sagittal/Coronal	Axial
Acquisition time per scan (minutes)	02:09	01:43

Data from the T_1_ mapping sequence were exported both as magnitude DICOM images and k-space data. The T_1_ map was generated from the k-space data, according to image reconstruction details in the next section, and then co-registered to the DICOM data, which served as the geometric reference, since the T_1_ map reconstruction did not include corrections for system imperfections. Data for the T_2_-weighted scans were only exported as magnitude DICOM data.

#### 2.1.2 Image processing

Data from all inversion times were incorporated into a joint reconstruction problem. Each voxel was modelled as exhibiting a single-component T_1_ recovery, resulting in an exponential signal evolution. To exploit the inherent redundancy along the T_1_ recovery dimension, a local low-rank constraint was imposed to regularize the iterative reconstruction process (Zhang et al., 2015). The corresponding optimization problem can be expressed as:

$$\left\{{\hat{x}}_{1,\;\ldots ,\;N}\right\}=\underset{x_{1,\;\ldots ,\;n}}{argmin}\sum_{n=1}^{N}\;||DFx_{n}-y_{n}\text{​}|{|}_{2}^{2}+\lambda \sum_{b\;\in \;\text{Ω}}\;||R_{b}\left\{x_{1,\;\ldots ,\;N}\right\}|{|}_{*},\;$$ (1)

where D represents the sampling mask, F the 3D FFT operator, $x_{n}$ the unknown images to be reconstructed, and $y_{n}$ the undersampled k-space data for each TI_n_ (n=1,2,…,N ). The operator $R_{b}$ extracted a small spatial 3D block around pixel-index b in image-space, vectorized each one and concatenated them along all vectorized arrays from all different TIs to form the Casorati matrix. The low-rank property of the data in the TI dimension is enforced by the nuclear norm $||\cdot |{|}_{*}$, equivalent to the L_1_ norm of the singular values of $R_{b}$, and the strength of the regularisation is controlled by λ. An Alternating Direction Method of Multipliers (ADMM) solver was used to reconstruct all TIs jointly with 20 iterations. Coil sensitivity maps were calculated from the TI = 999 ms image using ENLIVE (Holme et al., 2019). All image reconstruction was performed with the BART toolbox (Wang et al., 2021), and detailed descriptions of reconstruction commands are found in the Supporting Information.

Prior to T_1_ fitting, phase correction was applied using the phase of the last TI as a reference to obtain the real-valued images (Bydder et al., 2002). T_1_ maps were fitted using the QUIT toolbox (Wood, 2018) from the real-valued data using the signal equation presented by Padormo et al. (2023). In addition to the T_1_-maps, the fitting procedure also calculated the proton density and the root mean squared error (RMSE) of the residuals. Brain masks for the T_1_ data were generated using HD-BET (Isensee et al., 2019).

The three T_2_-weighted images were combined into an isotropic volume (1.6 × 1.6 × 1.6 mm^3^) using the method described by Deoni et al. (2022), which was then used for automatic segmentation of brain structures with Samseg (Puonti et al., 2016). The segmentation labels were warped to the native space of the T_1_ maps using ANTs (Tustison et al., 2021). Details about the segmentation and registration process are described in the Supporting Information.

To visualise the average T_1_ values across all participants, the fitted T_1_ maps from each site were warped to a common template space (ICBM 2009a Nonlinear Symmetric, Fonov et al., 2011) using a deformable transformation (Tustison et al., 2021). Due to data-sharing constraints, the mean and standard deviation were calculated on a site-by-site basis across all participants. The mean T_1_ maps from each site were combined to create a pooled average and a pooled standard deviation map. The pooled average T_1_ map represented the typical T_1_ across the brain regions in the cohort, whereas the pooled standard deviation T_1_ map highlighted regions with greater inter-subject variability.

The image processing for the phantom data was identical to that used for in vivo data, with the exception of the segmentation stage. Each NiCl_2_ sphere in the phantom was automatically segmented by co-registering the phantom data to a template with Regions-Of-Interest (ROIs) provided for each compartment.

### 2.2 Study design

#### 2.2.1 Reproducibility in phantoms across all sites

Prior to the in vivo study, all sites acquired phantom data using the in vivo protocol on 3 consecutive days. Given that all sites were equipped with identical scanners and phantoms, the objective was to assess the reproducibility of the T_1_-mapping method across different sites, accounting for potential variations in noise levels, which could impact the results. Additionally, the phantom study was used to test the pipeline at different sites to ensure its functionality and consistency before applying it to in vivo data. For each of the NiCl_2_ spheres in the phantom, the mean and standard deviation of T_1_ were calculated. To assess the reproducibility of the method across different sites, the mean values of two spheres close to the expected T_1_ values of white matter (WM, 255 ms) and grey matter (GM, 377 ms) were employed (Jordanova et al., 2023). A one-way ANOVA (Analysis of Variance) was used to determine whether there were statistically significant differences in T_1_ between the sites, with a significance threshold of α=0.05.

#### 2.2.2 Longitudinal repeatability in phantom and in vivo in one site

To evaluate the longitudinal repeatability of the measurements, a single subject was scanned six times at one site over a period of 2 months, with phantom data also collected prior to each scan. For the phantom data, the same two spheres as in Part 1 were chosen as regions of interest (ROIs), each containing approximately 30 voxels. For the in vivo data, the cerebral white matter (WM), cortical gray matter (GM), and the caudate were included. The WM and GM ROIs each contained approximately 9000 voxels, while the caudate—added as the smallest segmented brain structure in our dataset—contained approximately 200 voxels. Including the caudate enabled a more size-matched comparison to the phantom ROIs and helped assess the potential influence of ROI size on repeatability metrics. To evaluate the repeatability over time, the Coefficient of Variation (CV) was calculated for each structure, whereby the temporal standard deviation was divided by the mean value. The repeatability coefficient (RC) was also calculated, representing the maximum expected difference among measurements for the same subject 95% of the time, as RC = 1.96 × √2× temporal SD.

#### 2.2.3 Repeatability and reproducibility in vivo across sites

Each site then proceeded to acquire in vivo data from 10 volunteers. For each volunteer, the T_1_ mapping protocol was acquired twice within the same session without repositioning, for test-retest purposes, except for one site where only one run of the T_1_ mapping protocol was collected. Brain segmentation masks were obtained from the T_2_w image, as described in the Image Processing section. The following bi-lateral ROIs obtained from Samseg were used: cerebral WM, cortical GM, caudate, cerebellum, putamen, and thalamus. For each segmented region, the mean T_1_ values were extracted. A one-way ANOVA test was used to check for significant differences in the mean WM and GM values between sites. Histograms for the WM and GM masks were also produced with 200 bins from 1 to 500 ms and their full width at half maximum (FWHM) and skewness were computed to assess the spread and asymmetry of the distributions, respectively. To evaluate whether the segmented brain structures could be differentiated by their T_1_ values, a one-way ANOVA was performed, followed by post hoc pairwise comparisons using Tukey’s Honest Significant Difference (HSD) test (Abdi, 2010). To assess the repeatability of the method, Bland-Altman plots were created to compare the T_1_ values from two consecutive runs, and the T_1_ values from ROIs in the left and right hemispheres (Bland & Altman, 1986).

## 3 Results

### 3.1 Reproducibility in phantom across all sites

For the phantom measurements, the longitudinal relaxation rate (R_1_ = 1/T_1_) was used for linear fitting, as the relationship between R_1_ and NiCl_2_ concentration is expected to be linear according to relaxation theory. This approach simplifies the fitting model and allows for direct estimation of the longitudinal relaxivity (r_1_).

**Fig. 1.**
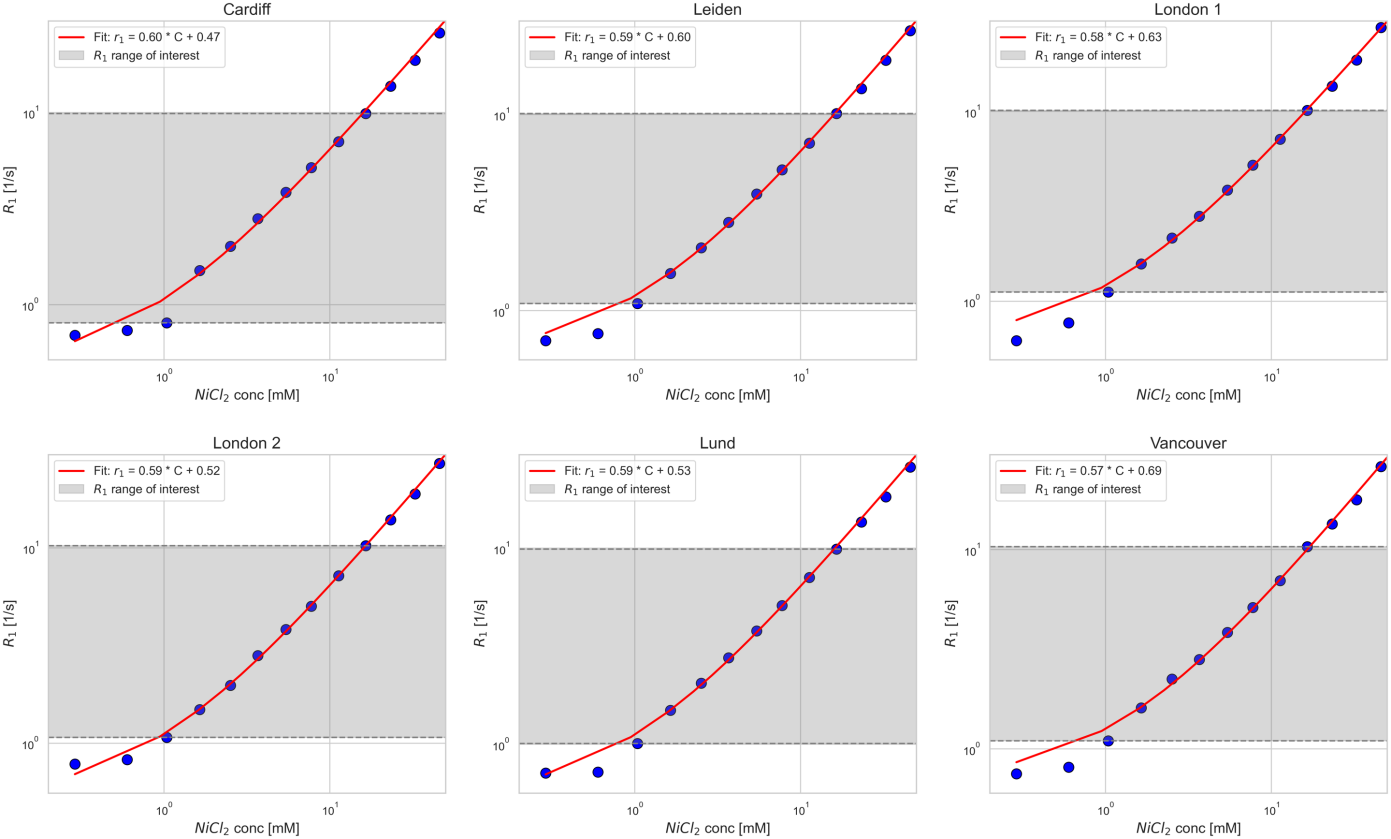
Relaxation rate R_1_ versus NiCl_2_ concentration in the phantom for all sites, with calculated longitudinal relaxivity *r_1_* as the red fitted line to the equation $\;R_{1}=r_{1}\cdot C+R_{1,C=0}$ , with *r_1_* being the slope, C the NiCl_2_ concentration, and *R_1,C=0_* the intercept equivalent to the relaxation rate without any NiCl_2_ doping. The relaxivity fit was limited to the grey region (T_1_ between 100 and 1000 ms) for which the T_1_ mapping protocol was optimized. The data are here shown with logarithmic axes to better visualize all spheres in the phantom.

The relationship between R_1_ and the NiCl_2_ concentration was found to be consistent across all six sites, with the calculated r_1_ derived from linear fits within the optimized T_1_ range (100–1000 ms, Fig. 1). The fitted relaxivity slope from each site was within 3% of the average slope, indicating a consistent level of T_1_ measurement accuracy across sites in our experimental protocol.

**Fig. 2.**
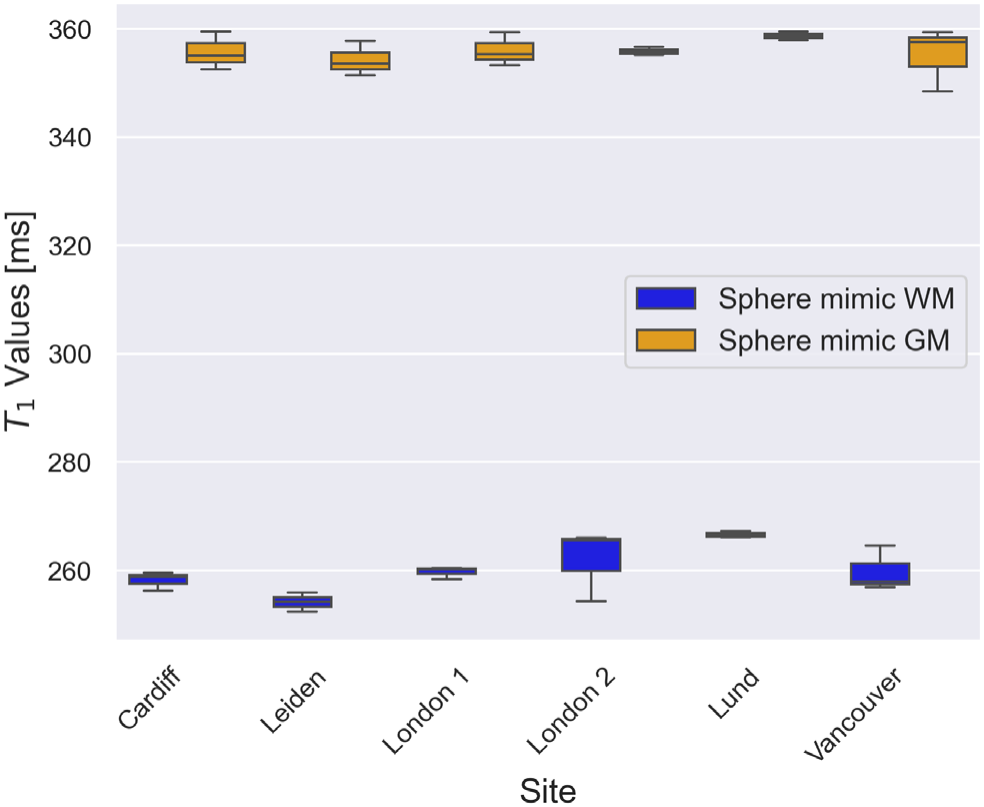
Average T_1_ values in two spheres mimicking white matter and cerebral cortex calculated from three phantom sessions at each site.

The calculated T_1_ values in spheres mimicking values found in white matter and cortex, derived from three phantom sessions at each site, demonstrated good inter-site consistency (Fig. 2). No significant inter-site difference was observed in T_1_ for the cortex mimic (p = 0.11, F = 2.28). More variations were present for the white matter mimic, with deviations ranging from 0.26% to 2.73% relative to the overall mean T_1_ for this mimic, but they were not statistically significant mimic (p = 0.09, F = 2.54).

A potential explanation for these variations is the sensitivity of relaxation times to temperature (Martin et al., 2023). The phantom scans at each site were conducted at slightly different temperatures (see Supplementary Table S3), which could have influenced the T_1_ values. Despite this, the observed variability remained small, which reflects the robustness of the protocol in standardised phantom conditions.

### 3.2 Longitudinal repeatability in phantoms and in vivo at one site

**Fig. 3.**
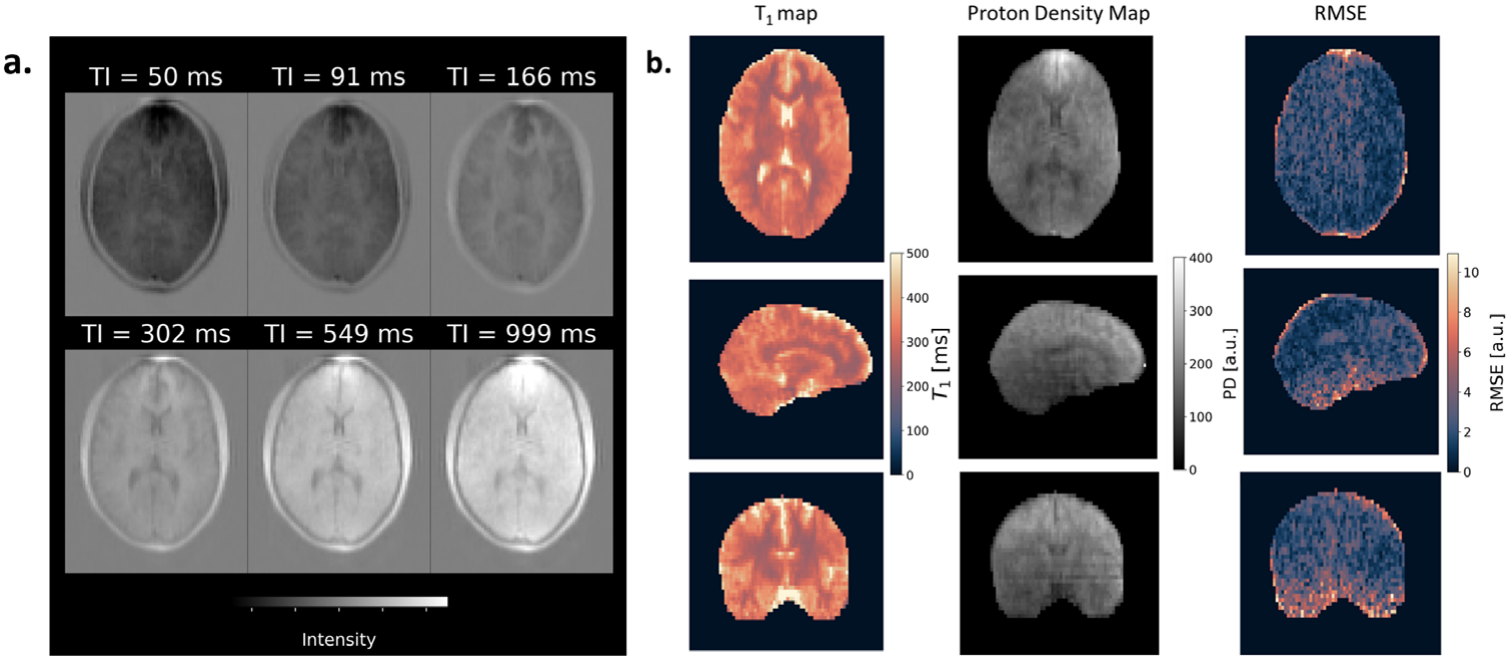
(a) Example of reconstructed real-valued images acquired with different inversion delay (TI) from a volunteer; (b) Calculated T_1_, Proton Density (PD) and root-mean-square error (RMSE) maps: the RMSE map showed that the model fit is equally good in both WM and cortex; T_1_ and PD estimates in cerebrospinal fluid were inaccurate, as expected, since the protocol was not optimised for long relaxation times.

Figure 3 illustrates an example of the real-valued images and T_1_, proton density, and RMSE maps from a single volunteer. The T_1_ map distinctly delineated the white matter and cortex boundaries across all three scan planes. The RMSE map showed uniform noise with no visible structure, suggesting that the model fit all tissue types equally well.

**Fig. 4.**
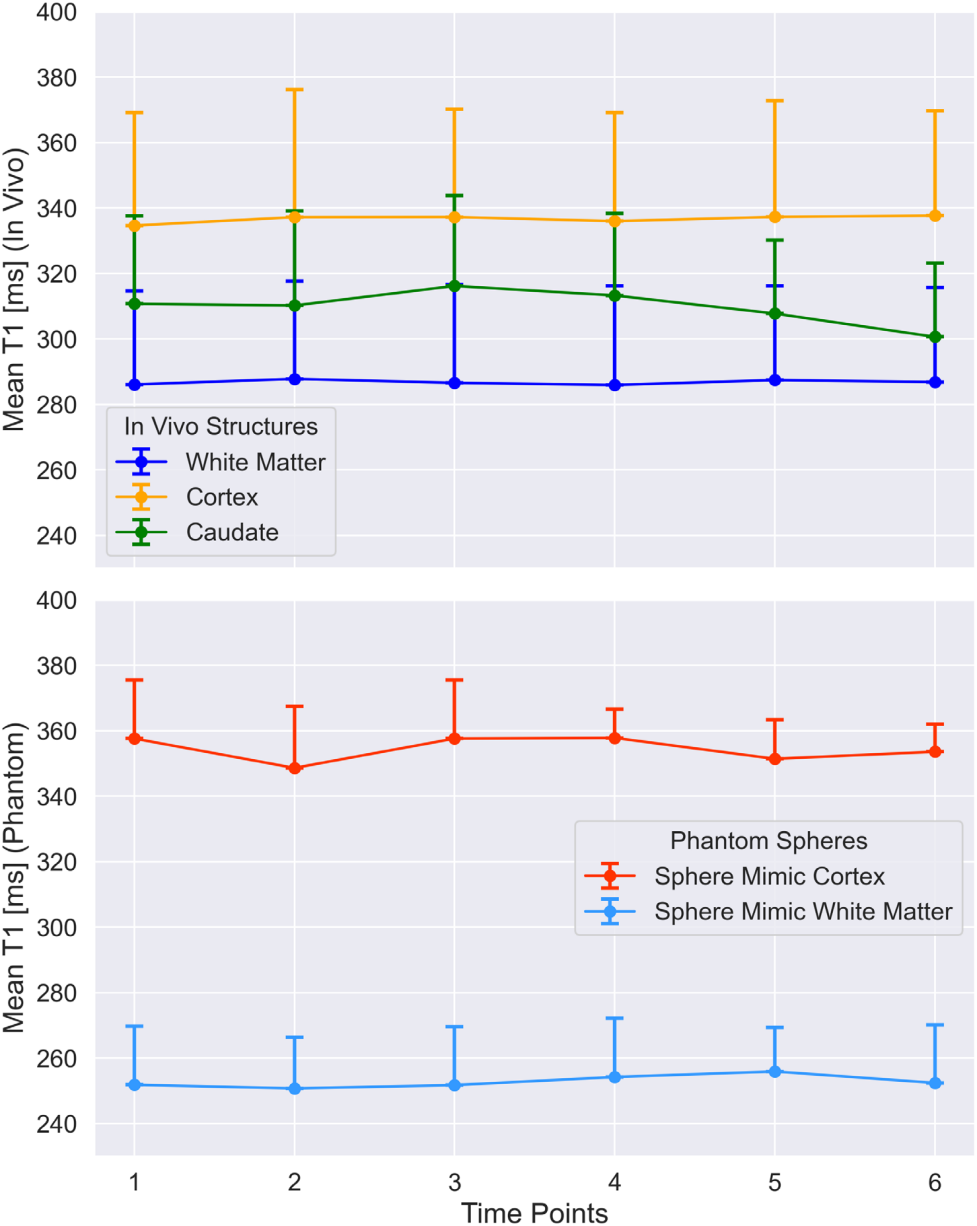
Average T_1_ values with one-sided SD error bars measured in the white matter, cerebral cortex, and caudate of one volunteer at six time points over a 2-month period, together with the phantom containing spheres mimicking the aforementioned tissues.

The longitudinal measurements demonstrated high repeatability over time both in vivo and in the phantom, with minimal variability (Fig. 4; Table 2). The mean T_1_ values for both white matter and cortical structures in vivo appeared stable across six time points over 2 months (RC < 5 ms), indicating that the protocol provides reliable and repeatable T_1_ measurements in these structures in vivo over time. Furthermore, the T_1_ measurement in the white matter demonstrated the lowest variability, as indicated by the lowest temporal SD, CV, and RC. The phantom measurements also showed consistent mean T_1_ values for the two spheres mimicking white matter and cortical grey matter. The coefficients of variation for in vivo (average 0.30%) and phantom (average 0.93%) measurements was similar, supporting the protocol’s robustness and repeatability under varying conditions.

**Table 2.** Summary of the statistical analysis of repeated T1 measurements in a single volunteer and two phantom spheres at one site.

Structure	Average T_1_ [ms]	Temporal σ(T_1_) [ms]	Coefficient of Variation [%]	Repeatability Coefficient [ms]
White Matter (in vivo)	286.8	0.7	0.26	2
Cerebral Cortex (in vivo)	336.7	1.2	0.35	3.2
Caudate (in vivo)	309.8	5.3	1.72	14.8
Sphere WM (phantom)	252.8	1.9	0.75	5.3
Sphere Cortex	354.4	3.9	1.1	10.8
(phantom)				

Reported metrics include the mean T1 value, temporal standard deviation, coefficient of variation, and repeatability coefficient.

To investigate the influence of ROI size on repeatability, the caudate—the smallest segmented brain structure (~200 voxels)—was included in the analysis. As expected, the smaller ROI showed slightly increased variability compared to larger ROIs, with a CV of 1.72% and an RC of 14.79 ms. However, this increase was modest and did not approach the higher RC values observed in the phantom, despite the phantom ROIs being even smaller (~30 voxels). This suggests that factors beyond ROI size, such as system-level or environmental variability, may contribute more substantially to reduced repeatability in the phantom measurements.

### 3.3 Repeatability and reproducibility in vivo across all sites

**Fig. 5.**
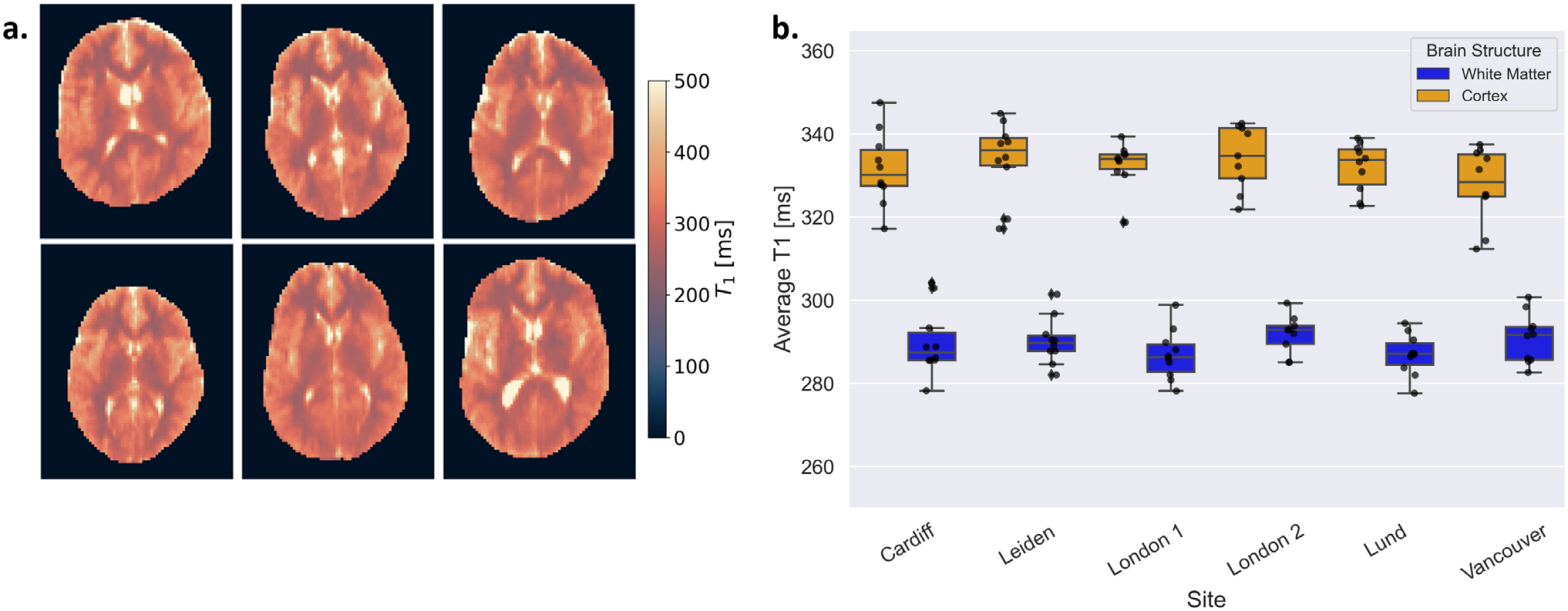
(a) Selected T_1_ maps across all volunteers, chosen to illustrate the variability observed within our dataset. (b) Boxplots showing the average T_1_ values for white matter and cerebral cortex across healthy subjects (n = 10) imaged at each site. The lower whisker extends from the first quartile (Q1) to the smallest data point within 1.5 times the interquartile range (IQR) below Q1, and the upper whisker extends from the third quartile (Q3) to the largest data point within 1.5 times the IQR above Q3. Data points outside the whiskers are considered outliers and plotted as individual diamond points (♦). Individual data points for each volunteer are overlaid as black dots (•) for better visualisation of variability within site.

Figure 5a shows T_1_-maps from each of the six sites to illustrate the similarity in the T_1_-maps despite differences in head size and shape, anatomical features, and positioning. The T_1_ values measured in white matter and cortex were highly consistent across sites (Fig. 5b). There were no significant differences in the T_1_ values between sites in neither white matter (p = 0.34) nor cerebral cortex (p = 0.47).

**Fig. 6.**
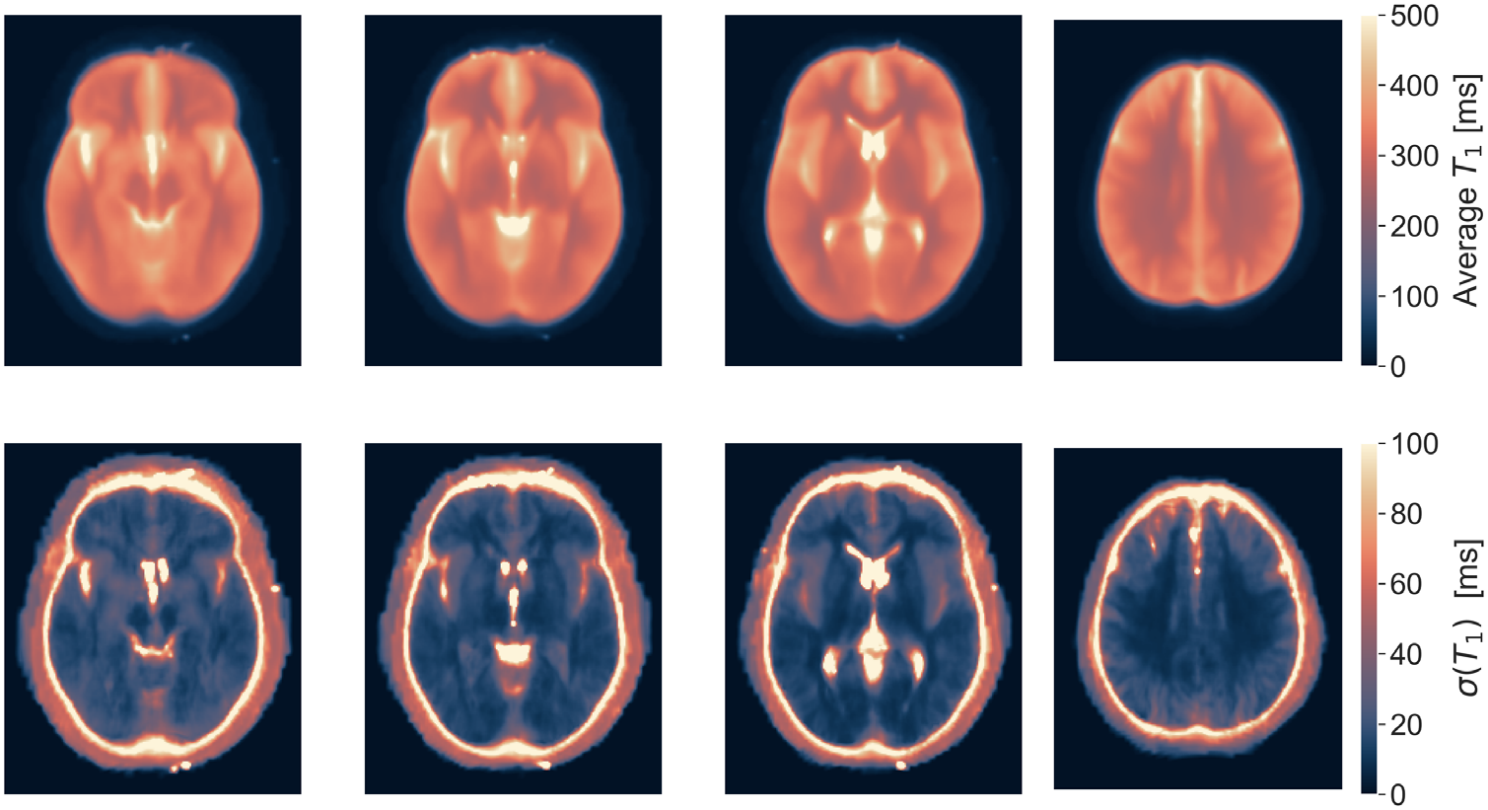
Representative slices from the pooled average and pooled standard deviation T_1_ maps across sites for all subjects (n = 60). Note that the colour scale range is different in the two maps.

The average ± standard deviation in vivo T_1_ values, pooled from all sites, was 290 ± 6 ms in WM and 332 ± 8 ms in the cortex (Fig. 6). The pooled average T_1_ map clearly delineated WM from the cortex and highlighted subcortical structures. The mean T_1_ values align with previously reported ranges (Jordanova et al., 2023; O’Reilly & Webb, 2022). The pooled standard deviation map showed low variability in WM, indicating consistent T_1_ measurements in this structure across the cohort. In contrast, higher variability in cerebrospinal fluid (CSF), as expected since the protocol was not optimized for long T_1_s, and in cortical grey matter, is attributed to variability in cortical folding leading to registration errors.

**Fig. 7.**
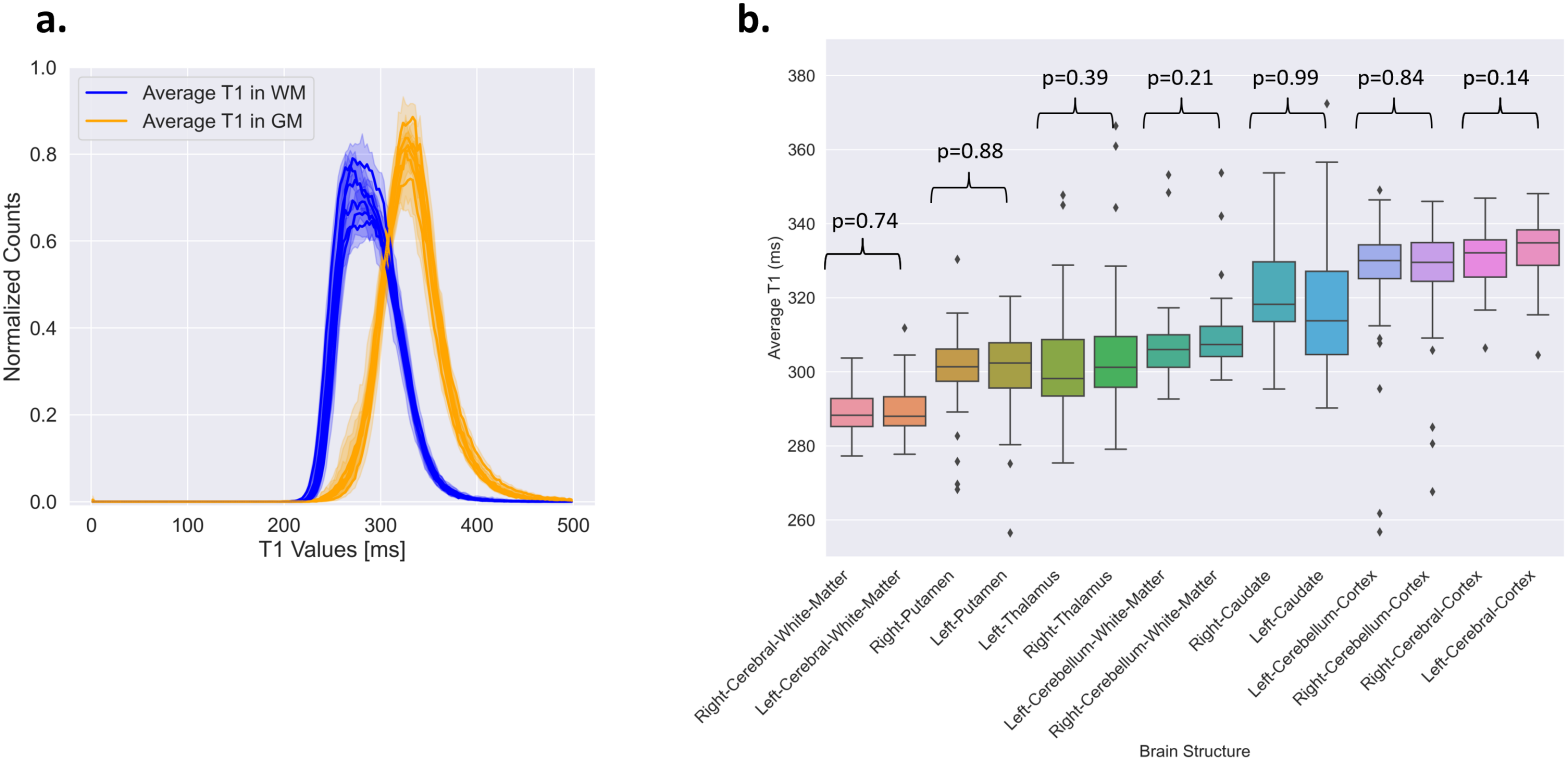
(a) Histograms of the T_1_ maps in white matter (WM) and cortex (GM) across the whole brain for all volunteers. The average distribution from each site is represented by a thick line, and with the standard deviation as shadowed line around it. (b) T_1_ values calculated using automatic segmentation of different brain structures in all subjects (n = 60). A comparison of T_1_ values between the right and left sides for each structure revealed no significant differences (p > 0.05).

The histograms presented in Figure 7a demonstrate distinct distributions of T_1_ values for white matter and cortex across all volunteers, with minimal inter-site variability.

The mean T_1_ values for GM were consistently higher than those for WM, in line with expectations. WM has a broader (FWHM = 0.0720 seconds) but more symmetric (skewness = 1.87) T_1_ distribution, suggesting more variability but less asymmetry. GM has a narrower (FWHM = 0.0612 seconds) but more skewed (skewness = 2.01) distribution, indicating that while T_1_ values are more tightly clustered, there are still some longer tails toward higher T_1_ values. These findings are consistent with expected tissue characteristics, as GM generally has higher and more variable T_1_ values than WM due to differences in water content and myelination. Differences between sites are small, which suggests that while some site-to-site variation exists, the overall trend is consistent across all locations (specifics about each site can be found in Supplementary S4).

Figure 7b shows the distribution of T_1_ values across brain structures for all participants, with the left and right hemispheres displayed separately. No significant hemispheric differences were observed for any structure (paired t-tests, all p > 0.05). Some smaller structures, like the thalamus and caudate nucleus, showed higher variability, likely due to partial volume effects, while larger structures such as white matter showed more consistent T_1_ values.

Moreover, T_1_ values differed significantly between brain structures, as confirmed by a one-way ANOVA (F = 82.99, p < 1e-10). Post hoc comparisons using Tukey’s HSD test showed that most structures had significantly different T_1_ values (details in Supplementary S5). However, a few structure pairs did not differ significantly: caudate versus cerebellar cortex (p = 0.30), cerebellar cortex versus cerebral cortex (p = 0.39), cerebellar white matter versus thalamus (p = 0.38), and putamen versus thalamus (p = 0.95). These results indicate that most brain structures can be distinguished based on their T_1_ values, although some overlap remains.

**Fig. 8.**
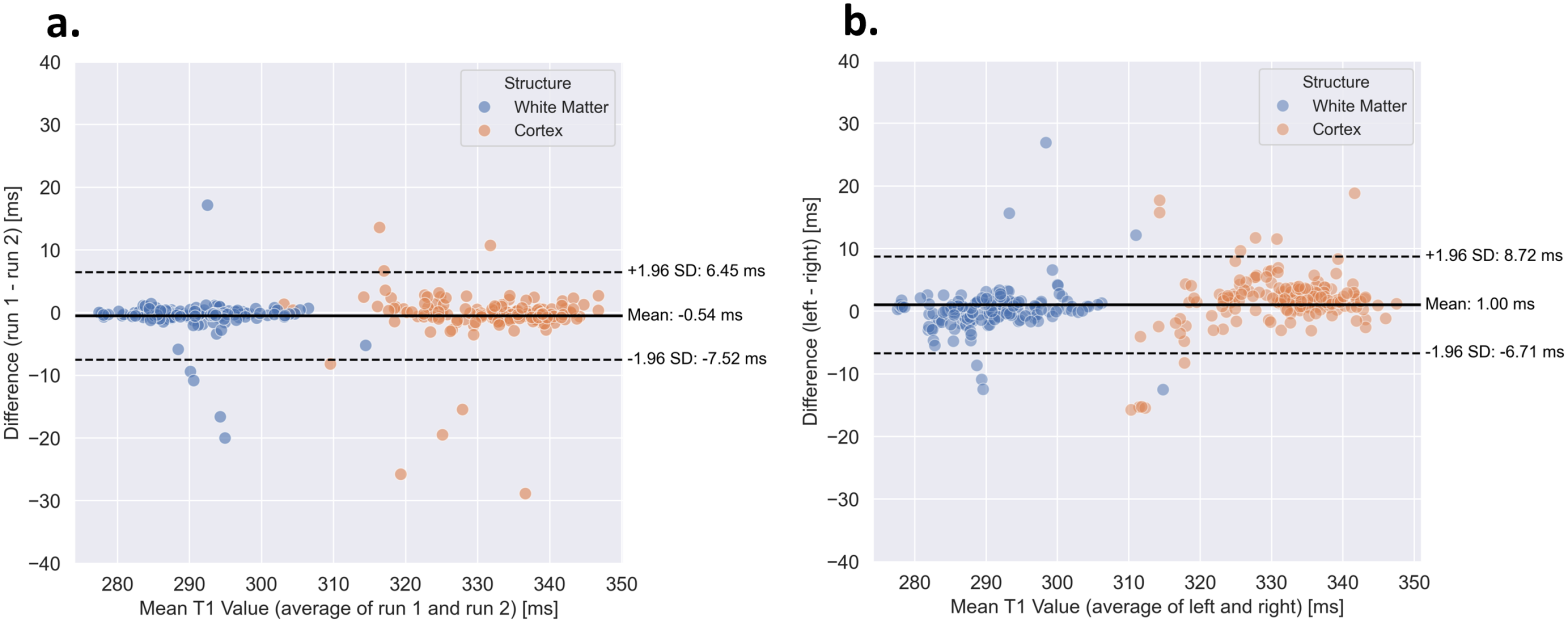
Bland-Altman plot of the T_1_ values measured in white matter and cortex for two consecutive runs (a) and for the left and right hemisphere (b), in all subjects (except London 1 that has only one run). Mean differences are marked as the black line, and ±1.96 standard deviations from the mean difference are indicated with dashed lines.

A Bland–Altman plot comparing runs 1 and 2 is presented in Figure 8, alongside a separate analysis examining potential left–right differences that could arise from RF coil asymmetries or B_0_ inhomogeneities. The T_1_ estimates derived from runs 1 and 2 exhibited a high degree of concordance for both white matter and cortical measurements (Fig. 8a), mean ± std difference of -0.59 ± 2.84 in WM and -0.48 ± 4.17 in cortex. While a few outliers were observed, a similar level of agreement was noted between the right and left hemispheres (Fig. 8b), mean ± std difference 0.16 ± 3.46 in WM and 1.85 ± 4.20 ms in cortex. These findings indicate that the scanner provides consistent T_1_ measurements across hemispheres, with no observable left-right bias in the data. Both RF coil properties and B0 inhomogeneities could, in principle, contribute to left–right bias. However, since our data show no such bias, these factors are unlikely to have introduced systematic hemispheric effects in our measurements. A summary of the T_1_ values obtained within the cohort is reported in Table 3.

**Table 3.** Summary for each site of the average T_1_ values [ms], the mean T_1_ differences [ms] between run and hemispheres, for both white matter and cerebral cortex.

	White Matter			Cerebral Cortex		
Site	Average	ΔT_1_/run	ΔT_1_/hemisphere	Average	ΔT_1_/run	ΔT_1_/hemisphere
Cardiff	290 ± 8	2	3	332 ± 9	<1	4
Leiden	290 ± 6	<1	4	334 ± 9	1	3
London 1	287 ± 6	-	4	333 ± 6	-	4
London 2	292 ± 5	<1	1	334 ± 8	<1	4
Lund	287 ± 5	<1	1	332 ± 6	<1	3
Vancouver	291 ± 7	1	4	328 ± 10	1	6

## 4 Discussion

The purpose of this study was to evaluate the reproducibility and repeatability of a 10-minute quantitative T_1_ acquisition at 64 mT. Quantitative phantoms were initially scanned with two objectives: first, to verify the correct implementation of the study protocol; second, to characterize the performance of each system. Subsequently, the phantom and one volunteer were scanned during multiple time points at a single site to evaluate longitudinal repeatability. Finally, 10 volunteers were scanned with a test-retest approach at each site. In all cases, a rapid T_1_ mapping protocol, with a total duration of approximately 10 minutes, was employed. The method was found to be highly repeatable, with a CoV below 2% both in phantom and in vivo. The method was also found to be highly reproducible: despite differences in temperature of the phantom and in age of the participants at each site, consistent estimates with minimal variation were observed both in the phantom (differences below 3% from the average) and in vivo (differences below 4% from the average).

This study is, to the authors’ knowledge, the first to conduct multi-site T_1_ mapping in vivo using low-field MRI, with a field strength of 64 mT. With a sample size of N = 60, we were able to provide normative values for T_1_ in WM and GM, as well as numerous subcortical structures. The close alignment of our T_1_ values with those reported by Jordanova et al. (2023) provides evidence of the reliability of very low field imaging for quantitative measurements. Discrepancies between studies are likely attributable to methodological variations, including those pertaining to the selection of regions of interest and pulse sequence design. To minimize user bias and ensure consistency across the dataset, we employed an automated segmentation approach. However, partial volume effects can introduce errors, particularly in cortical grey matter regions where cerebrospinal fluid may influence measurements. While these effects are inherent to MRI (Gonzalez Ballester et al., 2002), they may be more pronounced at very low field due to lower SNR and spatial resolution (2.5 x 2.5 x 3.5 mm³).

The reproducibility and repeatability of quantitative T_1_ mapping have been extensively investigated at clinical field strengths, particularly at 1.5 T and 3 T, with reported coefficients of variation typically ranging between 2 and 5% in healthy brain tissue, depending on acquisition method and processing pipeline (Leutritz et al., 2020; Weiskopf et al., 2013). Several multicentre studies have established benchmarks for such measurements, highlighting the influence of scanner vendor, sequence choice, and field strength on inter-site variability (Bane et al., 2018). For example, at 7 T, a study reported a CoV of ~5% across sites despite identical vendor platforms, underscoring the role of hardware and software differences (Voelker et al., 2021). At 3 T, a dual-vendor multiparameter mapping study across six sites found intra- and inter-site CoVs between 4–10% for parameters such as R_1_, proton density, and magnetisation transfer saturation (Leutritz et al., 2020), while multicentre phantom studies at 1.5 T and 3 T reported repeatability between 0.2 and 8.3% (Keenan et al., 2021). In this context, the CoVs below 2% achieved in the present study at 64 mT are comparable to those reported at higher field strengths, despite the more challenging SNR and hardware conditions of very-low-field MRI. These findings provide initial evidence that reproducible multi-site T_1_ mapping is achievable even at low field, where reproducibility metrics remain largely unexplored.

The high degree of reproducibility observed between sites is likely attributable to the near-identical operating conditions present at each site. The same scanner models were employed at each site, operating at the same software level, and utilising the same pulse sequence for data acquisition. These findings reinforce the necessity for harmonised imaging parameters, a conclusion corroborated by prior studies on T_1_ mapping reproducibility (Bane et al., 2018; Keenan et al., 2025; Yen et al., 2023), such as the ISMRM study (Boudreau et al., 2024). Our results demonstrate that consistent inter-site reproducibility can be achieved even at very low field strengths, thereby offering new opportunities for multi-site research in resource-limited settings or portable imaging applications.

While T_1_ in white and grey matter is significantly shorter at 64 mT compared to 3 T, the T_1_ of cerebrospinal fluid remains relatively constant across field strengths (Rooney et al., 2007). Consequently, the larger difference between T_1_ in white and grey matter versus CSF complicates the accurate mapping of all three tissues using a single protocol. Therefore, we focused on quantifying T_1_ in white and grey matter, as accurately mapping CSF would require longer TR and TI, thus increasing the acquisition time greatly.

In this study, we did not implement retrospective motion correction between the different inversion times. This was primarily due to the absence of discernible motion during the acquisition process. Properly accounting for motion between TIs is difficult when employing a reconstruction framework with regularisation in the TI-dimension. This would either require a reconstruction method with joint regularisation and motion correction, or a two-step approach where motion parameters are estimated first, and then k-space data are corrected prior to reconstruction.

There are numerous approaches for joint reconstruction of multi-contrast data for quantitative parameter mapping. The low-rank constraint used in this work was selected due to its relatively fast processing time, lack of imposition of a specific signal model in the reconstruction, and ready availability in the BART toolbox. Alternative approaches, such as model-based methods, could also be explored further, either as a subspace approach (Dvorak et al., 2023) or non-linear inversion of the Bloch equations (Scholand et al., 2023). Both low-rank and the other approaches would benefit from an increase in the number of time points acquired, with each TI being acquired with a higher undersampling factor.

This work is conducted as a part of the global UNITY consortium (Ultra-low field Neuroimaging In The Young), which aims to advance paediatric neuroimaging using ultra-low-field MRI to improve access to brain health assessments, particularly in low-resource settings, and enhance understanding of infant neurodevelopment (Abate et al., 2024). T_1_ mapping offers insights into brain development, disease progression, and the optimisation of novel clinical applications. Within the UNITY consortium (Abate et al., 2024), T_1_ is of particular significance as it can be used to track myelination, with higher myelin content being reflected by lower T_1_, markedly during the first 3 months of life, before stabilising in adolescence (Dong et al., 2023; Steen et al., 1995). Although the present study has been conducted in adults, the T_1_ mapping protocol presented has been optimised for a broad range of T_1_ values up to approximately 1000 ms. To adapt the protocol for tissues with longer T_1_ values, such as those observed in neonatal brain imaging (e.g., in frontal white matter, where T_1_ values can exceed 1000 ms (Padormo et al., 2023)), the inversion time range would need to be extended, which would consequently increase the repetition time, leading to longer scan durations. Since this is less desirable in neonatal imaging due to the potential for motion, scan time could be reduced by optimising the partial Fourier factor or increasing the acceleration factor, while tailoring the Field-Of-View to smaller head sizes. Importantly, even relatively small in vivo structures such as the caudate (~200 voxels) demonstrated good repeatability (RC ≈ 15 ms), with only modestly increased variability compared to larger ROIs. In contrast, the phantom—despite its lower biological variability—showed higher RC values, suggesting that external influences (e.g., scanner stability, temperature fluctuations) may dominate repeatability performance when measuring very small, homogeneous objects. These observations are particularly relevant for neonatal imaging, where brain structures are smaller and segmentation is more challenging. While ROI size may contribute to increased variability, system-level factors must also be considered when interpreting repeatability in small structures. Such adaptations would align with the UNITY consortium’s focus on early-life brain imaging.

Although the scans acquired in this study were performed in unshielded rooms, these were dedicated imaging spaces without active clinical equipment. Future studies should assess system performance in true Point-of-Care environments, such as patient rooms or emergency settings, to validate its robustness amid additional EMI sources.

## 5 Conclusion

We have demonstrated a protocol for in vivo T_1_ mapping of the adult brain using a portable, 64 mT, MRI system with an acquisition time of 10 minutes. The protocol was evaluated in a multi-centre study with 60 healthy volunteers, with T_1_ values in white and grey matter showing high reproducibility between sites and high repeatability within sites. Robustness of the protocol was further validated by phantom measurements at each site and a longitudinal study in a single site. These results show that T_1_ mapping can be performed with robust results at 64 mT, opening up the possibility of employing T_1_ as a biomarker for clinical and neuroscience studies using portable MRI systems.

## Supplementary Material

Supplementary Material

## Data Availability

The .csv files from different sites, containing T_1_ values measured in various brain structures collected during this project, are shared. However, due to privacy concerns, the in-vivo images cannot be made available. Tabular data and code for reproducing (most of) the figures in the paper can be found here: https://github.com/UNITY-Physics/unity_t1mapping_paper The reconstruction and image processing steps with BART toolbox are described in the Supplementary S2.

## References

[IMAG.a.916-b1] Abate, F., Adu-Amankwah, A., Ae-Ngibise, K. A., Agbokey, F., Agyemang, V. A., Agyemang, C. T., Akgun, C., Ametepe, J., Arichi, T., Asante, K. P., Balaji, S., Baljer, L., Basser, P. J., Beauchemin, J., Bennallick, C., Berhane, Y., Boateng-Mensah, Y., Bourke, N. J., Bradford, L., … Williams, S. (2024). UNITY: A low-field magnetic resonance neuroimaging initiative to characterize neurodevelopment in low and middle-income settings. Dev Cogn Neurosci, 69, 101397. 10.1016/j.dcn.2024.101397 39029330 PMC11315107

[IMAG.a.916-b2] Abdi, H. W., Lynne J. (2010). Tukey’s Honestly Signicant Difference (HSD) Test. In N. Salkind (Ed.), Encyclopedia of Research Design. Sage. 10.4135/9781412961288.n181

[IMAG.a.916-b3] Arnold, T. C., Tu, D., Okar, S. V., Nair, G., By, S., Kawatra, K. D., Robert-Fitzgerald, T. E., Desiderio, L. M., Schindler, M. K., Shinohara, R. T., Reich, D. S., & Stein, J. M. (2022). Sensitivity of portable low-field magnetic resonance imaging for multiple sclerosis lesions. Neuroimage Clin, 35, 103101. 10.1016/j.nicl.2022.103101 35792417 PMC9421456

[IMAG.a.916-b4] Bane, O., Hectors, S. J., Wagner, M., Arlinghaus, L. L., Aryal, M. P., Cao, Y., Chenevert, T. L., Fennessy, F., Huang, W., Hylton, N. M., Kalpathy-Cramer, J., Keenan, K. E., Malyarenko, D. I., Mulkern, R. V., Newitt, D. C., Russek, S. E., Stupic, K. F., Tudorica, A., Wilmes, L. J., … Taouli, B. (2018). Accuracy, repeatability, and interplatform reproducibility of T(1) quantification methods used for DCE-MRI: Results from a multicenter phantom study. Magn Reson Med, 79(5), 2564–2575. 10.1002/mrm.26903 28913930 PMC5821553

[IMAG.a.916-b5] Bland, J. M., & Altman, D. G. (1986). Statistical methods for assessing agreement between two methods of clinical measurement. Lancet, 1(8476), 307–310. 10.1016/s0140-6736(86)90837-8 2868172

[IMAG.a.916-b6] Boudreau, M., Karakuzu, A., Cohen-Adad, J., Bozkurt, E., Carr, M., Castellaro, M., Concha, L., Doneva, M., Dual, S. A., Ensworth, A., Foias, A., Fortier, V., Gabr, R. E., Gilbert, G., Glide-Hurst, C. K., Grech-Sollars, M., Hu, S., Jalnefjord, O., Jovicich, J., … ISMRM Reproducible Research Study Group and the ISMRM Quantitative MR Study Group. (2024). Repeat it without me: Crowdsourcing the T(1) mapping common ground via the ISMRM reproducibility challenge. Magn Reson Med, 92(3), 1115–1127. 10.1002/mrm.30111 38730562

[IMAG.a.916-b7] Bydder, M., Larkman, D. J., & Hajnal, J. V. (2002). Combination of signals from array coils using image-based estimation of coil sensitivity profiles. Magn Reson Med, 47(3), 539–548. 10.1002/mrm.10092 11870841

[IMAG.a.916-b8] Deoni, S. C. L., Dean, D. C., 3rd, O’Muircheartaigh, J., Dirks, H., & Jerskey, B. A. (2012). Investigating white matter development in infancy and early childhood using myelin water faction and relaxation time mapping. Neuroimage, 63(3), 1038–1053. 10.1016/j.neuroimage.2012.07.037 22884937 PMC3711836

[IMAG.a.916-b9] Deoni, S. C. L., O’Muircheartaigh, J., Ljungberg, E., Huentelman, M., & Williams, S. C. R. (2022). Simultaneous high-resolution T(2) -weighted imaging and quantitative T(2) mapping at low magnetic field strengths using a multiple TE and multi-orientation acquisition approach. Magn Reson Med, 88(3), 1273–1281. 10.1002/mrm.29273 35553454 PMC9322579

[IMAG.a.916-b10] Dong, Y., Deng, X., Xie, M., Yu, L., Qian, L., Chen, G., Zhang, Y., Tang, Y., Zhou, Z., & Long, L. (2023). Gestational age-related changes in relaxation times of neonatal brain by quantitative synthetic magnetic resonance imaging. Brain Behav, 13(7), e3068. 10.1002/brb3.3068 37248768 PMC10338790

[IMAG.a.916-b11] Dvorak, A. V., Kumar, D., Zhang, J., Gilbert, G., Balaji, S., Wiley, N., Laule, C., Moore, G. R. W., MacKay, A. L., & Kolind, S. H. (2023). The CALIPR framework for highly accelerated myelin water imaging with improved precision and sensitivity. Sci Adv, 9(44), eadh9853. 10.1126/sciadv.adh9853 37910622 PMC10619933

[IMAG.a.916-b12] Fatouros, P. P., Marmarou, A., Kraft, K. A., Inao, S., & Schwarz, F. P. (1991). In vivo brain water determination by T1 measurements: Effect of total water content, hydration fraction, and field strength. Magn Reson Med, 17(2), 402–413. 10.1002/mrm.1910170212 2062213

[IMAG.a.916-b13] Fellner, C., Nickel, M. D., Kannengiesser, S., Verloh, N., Stroszczynski, C., Haimerl, M., & Luerken, L. (2023). Water-fat separated T1 mapping in the liver and correlation to hepatic fat fraction. Diagnostics (Basel), 13(2), 201. 10.3390/diagnostics13020201 36673011 PMC9858222

[IMAG.a.916-b14] Fonov, V., Evans, A. C., Botteron, K., Almli, C. R., McKinstry, R. C., Collins, D. L., & Brain Development Cooperative Group. (2011). Unbiased average age-appropriate atlases for pediatric studies. Neuroimage, 54(1), 313–327. 10.1016/j.neuroimage.2010.07.033 20656036 PMC2962759

[IMAG.a.916-b15] Gonzalez Ballester, M. A., Zisserman, A. P., & Brady, M. (2002). Estimation of the partial volume effect in MRI. Med Image Anal, 6(4), 389–405. 10.1016/s1361-8415(02)00061-0 12494949

[IMAG.a.916-b16] Holme, H. C. M., Rosenzweig, S., Ong, F., Wilke, R. N., Lustig, M., & Uecker, M. (2019). ENLIVE: An efficient nonlinear method for calibrationless and robust parallel imaging. Sci Rep, 9(1), 3034. 10.1038/s41598-019-39888-7 30816312 PMC6395635

[IMAG.a.916-b17] Iglesias, J. E., Schleicher, R., Laguna, S., Billot, B., Schaefer, P., McKaig, B., Goldstein, J. N., Sheth, K. N., Rosen, M. S., & Kimberly, W. T. (2023). Quantitative brain morphometry of portable low-field-strength mri using super-resolution machine learning. Radiology, 306(3), e220522. 10.1148/radiol.220522 36346311 PMC9968773

[IMAG.a.916-b18] Isensee, F., Schell, M., Pflueger, I., Brugnara, G., Bonekamp, D., Neuberger, U., Wick, A., Schlemmer, H. P., Heiland, S., Wick, W., Bendszus, M., Maier-Hein, K. H., & Kickingereder, P. (2019). Automated brain extraction of multisequence MRI using artificial neural networks. Hum Brain Mapp, 40(17), 4952–4964. 10.1002/hbm.24750 31403237 PMC6865732

[IMAG.a.916-b19] Jordanova, K. V., Martin, M. N., Ogier, S. E., Poorman, M. E., & Keenan, K. E. (2023). In vivo quantitative MRI: T(1) and T(2) measurements of the human brain at 0.064 T. MAGMA, 36(3), 487–498. 10.1007/s10334-023-01095-x 37208553 PMC10386946

[IMAG.a.916-b20] Keenan, K. E., Gimbutas, Z., Dienstfrey, A., Stupic, K. F., Boss, M. A., Russek, S. E., Chenevert, T. L., Prasad, P. V., Guo, J., Reddick, W. E., Cecil, K. M., Shukla-Dave, A., Aramburu Nunez, D., Shridhar Konar, A., Liu, M. Z., Jambawalikar, S. R., Schwartz, L. H., Zheng, J., Hu, P., & Jackson, E. F. (2021). Multi-site, multi-platform comparison of MRI T1 measurement using the system phantom. PLoS One, 16(6), e0252966. 10.1371/journal.pone.0252966 34191819 PMC8244851

[IMAG.a.916-b21] Keenan, K. E., Tasdelen, B., Javed, A., Ramasawmy, R., Rizzo, R., Martin, M. N., Stupic, K. F., Seiberlich, N., Campbell-Washburn, A. E., & Nayak, K. S. (2025). T1 and T2 measurements across multiple 0.55T MRI systems using open-source vendor-neutral sequences. Magn Reson Med, 93(1), 289–300. 10.1002/mrm.30281 39219179 PMC11518643

[IMAG.a.916-b22] Lena, B., Teixeira, R. P., Padormo, F., Dong, Y., Sundgren, P. C., Webb, A., & Ljungberg, E. (2024). Fast and pseudo-random: Optimization of settings for rapid quantification of T1 in white and grey matter at 64 mT. Proc Intl Soc Mag Reson Med, 32, 2692. 10.58530/2024/2692

[IMAG.a.916-b23] Leutritz, T., Seif, M., Helms, G., Samson, R. S., Curt, A., Freund, P., & Weiskopf, N. (2020). Multiparameter mapping of relaxation (R1, R2*), proton density and magnetization transfer saturation at 3 T: A multicenter dual-vendor reproducibility and repeatability study. Hum Brain Mapp, 41(15), 4232–4247. 10.1002/hbm.25122 32639104 PMC7502832

[IMAG.a.916-b24] Ljungberg, E., Padormo, F., Poorman, M., Clemensson, P., Bourke, N., Evans, J. C., Gholam, J., Vavasour, I., Kollind, S. H., Lafayette, S. L., Bennallick, C., Donald, K. A., Bradford, L. E., Lena, B., Vokhiwa, M., Shama, T., Siew, J., Sekoli, L., van Rensburg, J., … Deoni, S. (2025). Characterization of portable ultra-low field MRI scanners for multi-center structural neuroimaging. Hum Brain Mapp, 46(8), e70217. 10.1002/hbm.70217 40405769 PMC12099222

[IMAG.a.916-b25] Martin, M. N., Jordanova, K. V., Kos, A. B., Russek, S. E., Keenan, K. E., & Stupic, K. F. (2023). Relaxation measurements of an MRI system phantom at low magnetic field strengths. MAGMA, 36(3), 477–485. 10.1007/s10334-023-01086-y 37209233 PMC10386925

[IMAG.a.916-b26] Mazurek, M. H., Cahn, B. A., Yuen, M. M., Prabhat, A. M., Chavva, I. R., Shah, J. T., Crawford, A. L., Welch, E. B., Rothberg, J., Sacolick, L., Poole, M., Wira, C., Matouk, C. C., Ward, A., Timario, N., Leasure, A., Beekman, R., Peng, T. J., Witsch, J., … Sheth, K. N. (2021). Portable, bedside, low-field magnetic resonance imaging for evaluation of intracerebral hemorrhage. Nat Commun, 12(1), 5119. 10.1038/s41467-021-25441-6 34433813 PMC8387402

[IMAG.a.916-b27] O’Muircheartaigh, J., Vavasour, I., Ljungberg, E., Li, D. K. B., Rauscher, A., Levesque, V., Garren, H., Clayton, D., Tam, R., Traboulsee, A., & Kolind, S. (2019). Quantitative neuroimaging measures of myelin in the healthy brain and in multiple sclerosis. Hum Brain Mapp, 40(7), 2104–2116. 10.1002/hbm.24510 30648315 PMC6590140

[IMAG.a.916-b28] O’Reilly, T., & Webb, A. G. (2022). In vivo T(1) and T(2) relaxation time maps of brain tissue, skeletal muscle, and lipid measured in healthy volunteers at 50 mT. Magn Reson Med, 87(2), 884–895. 10.1002/mrm.29009 34520068 PMC9292835

[IMAG.a.916-b29] Padormo, F., Cawley, P., Dillon, L., Hughes, E., Almalbis, J., Robinson, J., Maggioni, A., Botella, M. F., Cromb, D., Price, A., Arlinghaus, L., Pitts, J., Luo, T., Zhang, D., Deoni, S. C. L., Williams, S., Malik, S., J, O. M., Counsell, S. J., … Hajnal, J. V. (2023). In vivo T(1) mapping of neonatal brain tissue at 64 mT. Magn Reson Med, 89(3), 1016–1025. 10.1002/mrm.29509 36372971 PMC10099617

[IMAG.a.916-b30] Puonti, O., Iglesias, J. E., & Van Leemput, K. (2016). Fast and sequence-adaptive whole-brain segmentation using parametric Bayesian modeling. Neuroimage, 143, 235–249. 10.1016/j.neuroimage.2016.09.011 27612647 PMC8117726

[IMAG.a.916-b31] Rooney, W. D., Johnson, G., Li, X., Cohen, E. R., Kim, S. G., Ugurbil, K., & Springer, C. S., Jr. (2007). Magnetic field and tissue dependencies of human brain longitudinal 1H2O relaxation in vivo. Magn Reson Med, 57(2), 308–318. 10.1002/mrm.21122 17260370

[IMAG.a.916-b32] Scholand, N., Wang, X., Roeloffs, V., Rosenzweig, S., & Uecker, M. (2023). Quantitative MRI by nonlinear inversion of the Bloch equations. Magn Reson Med, 90(2), 520–538. 10.1002/mrm.29664 37093980

[IMAG.a.916-b33] Srinivas, S. A., Cauley, S. F., Stockmann, J. P., Sappo, C. R., Vaughn, C. E., Wald, L. L., Grissom, W. A., & Cooley, C. Z. (2022). External Dynamic InTerference Estimation and Removal (EDITER) for low field MRI. Magn Reson Med, 87(2), 614–628. 10.1002/mrm.28992 34480778 PMC8920578

[IMAG.a.916-b34] Steen, R. G., Gronemeyer, S. A., & Taylor, J. S. (1995). Age-related changes in proton T1 values of normal human brain. J Magn Reson Imaging, 5(1), 43–48. 10.1002/jmri.1880050111 7696808

[IMAG.a.916-b35] Stupic, K. F., Ainslie, M., Boss, M. A., Charles, C., Dienstfrey, A. M., Evelhoch, J. L., Finn, P., Gimbutas, Z., Gunter, J. L., Hill, D. L. G., Jack, C. R., Jackson, E. F., Karaulanov, T., Keenan, K. E., Liu, G., Martin, M. N., Prasad, P. V., Rentz, N. S., Yuan, C., & Russek, S. E. (2021). A standard system phantom for magnetic resonance imaging. Magn Reson Med, 86(3), 1194–1211. 10.1002/mrm.28779 33847012 PMC8252537

[IMAG.a.916-b36] Taylor, A. J., Salerno, M., Dharmakumar, R., & Jerosch-Herold, M. (2016). T1 mapping: Basic techniques and clinical applications. JACC Cardiovasc Imaging, 9(1), 67–81. 10.1016/j.jcmg.2015.11.005 26762877

[IMAG.a.916-b37] Tustison, N. J., Cook, P. A., Holbrook, A. J., Johnson, H. J., Muschelli, J., Devenyi, G. A., Duda, J. T., Das, S. R., Cullen, N. C., Gillen, D. L., Yassa, M. A., Stone, J. R., Gee, J. C., & Avants, B. B. (2021). The ANTsX ecosystem for quantitative biological and medical imaging. Sci Rep, 11(1), 9068. 10.1038/s41598-021-87564-6 33907199 PMC8079440

[IMAG.a.916-b38] Voelker, M. N., Kraff, O., Goerke, S., Laun, F. B., Hanspach, J., Pine, K. J., Ehses, P., Zaiss, M., Liebert, A., Straub, S., Eckstein, K., Robinson, S., Nagel, A. N., Stefanescu, M. R., Wollrab, A., Klix, S., Felder, J., Hock, M., Bosch, D., … Quick, H. H. (2021). The traveling heads 2.0: Multicenter reproducibility of quantitative imaging methods at 7 Tesla. Neuroimage, 232, 117910. 10.1016/j.neuroimage.2021.117910 33647497

[IMAG.a.916-b39] Wang, X., Rosenzweig, S., Scholand, N., Holme, H. C. M., & Uecker, M. (2021). Model-based reconstruction for simultaneous multi-slice T1 mapping using single-shot inversion-recovery radial FLASH. Magn Reson Med, 85(3), 1258–1271. 10.1002/mrm.28497 32936487 PMC10409492

[IMAG.a.916-b40] Wang, X., Wang, D., Li, X., Wang, W., Gao, P., Lou, B., Pfeuffer, J., Zhang, X., Zhu, J., Li, C., & Chen, M. (2022). A diagnostic index based on pseudo-continuous arterial spin labeling and T1-mapping improves efficacy in discriminating Alzheimer’s disease from normal cognition. Front Neurosci, 16, 974651. 10.3389/fnins.2022.974651 35992919 PMC9389211

[IMAG.a.916-b41] Weiskopf, N., Suckling, J., Williams, G., Correia, M. M., Inkster, B., Tait, R., Ooi, C., Bullmore, E. T., & Lutti, A. (2013). Quantitative multi-parameter mapping of R1, PD(*), MT, and R2(*) at 3T: A multi-center validation. Front Neurosci, 7, 95. 10.3389/fnins.2013.00095 23772204 PMC3677134

[IMAG.a.916-b42] Wood, T. C. (2018). QUIT: QUantitative Imaging Tools. J Open Source Softw, 3(26), 656. 10.21105/joss.00656

[IMAG.a.916-b43] Wu, J., Shi, Z., Zhang, Y., Yan, J., Shang, F., Wang, Y., Lu, H., Gu, H., Dou, W., Wang, X., & Yuan, L. (2021). Native T1 mapping in assessing kidney fibrosis for patients with chronic glomerulonephritis. Front Med (Lausanne), 8, 772326. 10.3389/fmed.2021.772326 34733870 PMC8558353

[IMAG.a.916-b44] Yen, Y. F., Stupic, K. F., Janicke, M. T., Greve, D. N., Mareyam, A., Stockmann, J., Polimeni, J. R., van der Kouwe, A., & Keenan, K. E. (2023). T1 relaxation time of ISMRM/NIST T1 phantom spheres at 7 T. NMR Biomed, 36(5), e4873. 10.1002/nbm.4873 36347826

[IMAG.a.916-b45] Zhang, T., Pauly, J. M., & Levesque, I. R. (2015). Accelerating parameter mapping with a locally low rank constraint. Magn Reson Med, 73(2), 655–661. 10.1002/mrm.25161 24500817 PMC4122652

