# Supplementary material for: Repeatability and reproducibility of rapid T1 mapping of brain tissues at 64 mT: A multicentre study
Source: Imaging Neurosci (Camb). 2025 Oct 8;3:IMAG.a.916. doi: 10.1162/IMAG.a.916 (PMC12508772; doi:10.1162/IMAG.a.916)
Supplement: Supplementary Material [file IMAG.a.916_supp.pdf]

Supporting Information

S1. Population demographics

Summary of the demographics from the sites are shown in Table S1.

***Table S1**: Summary demographics from each site.*

Site	Subjects (n)	Average Age (years)
Cardiff	10	29 (range 23-35)
Leiden	10	26 (range 20-30)
London 1	10	33 (range 25-43)
London 2	10	31 (range 24-47)
Lund	10	35 (range 19-51)
Vancouver	10	33 (range 25-54)

S2. Image Processing

The following sections describes further details of the analysis pipeline for image processing. When code snippets are shown `$` indicate bash commands and `>>>` indicate python commands.

Image reconstruction for T_1 mapping

Since the tools used for reading and processing the k-space data from the Hyperfine Swoop are proprietary material, we are only able to share general principles of the reconstruction pipeline. In the commands below, X:Y:Z indicate the matrix size. Reconstruction was performed using the bart python interface, but the shell commands are reported here for simplicity.

Sensitivity maps were estimated from the longest TI (999 ms) using ENLIVE with the nlinv command with the following options:

```
$ bart nlinv -m1 -d3 -a240 -b40 -R3 -i8 -x X:Y:Z -t
```

Using the ENLIVE sensitivity maps we run the iterative reconstruction with LLR regularization using the pics command:

```
$ bart pics -d 3 -e -S -N -d 4 -R L:7:7:0.004 -i 20 -b 4 -U -t traj -B basis data
sense pics_output
```

After running the pics command, we have a complex valued 4D dataset (X, Y, Z, TI). To obtained real valued data for T_1 mapping we employ the phase correction method presented by Bydder et al. [23]. We used the TI=999 ms as our phase reference with the assumption that the signal in white and grey matter then has recovered beyond the zero-crossing after the inversion pulse. Any phase variation in the image is then due to the object and not the T_1 recovery. We smooth this image with a Gaussian kernel with sigma=3, separately for the real and imaginary part, and divide the complex valued data by the magnitude to obtain a pure phase image. Each TI is then multiplied by the conjugate of this phase reference image, and we keep the real part for the T_1 fitting.

T_2w Isotropic reconstruction

The three T_2w images are combined into a single 1.6 mm isotropic resolution image using an interpolation method similar to [26]. Here we describe the main steps we used to obtain the isotropic image. The registration and interpolation steps were performed using ANTs[28].

1. All three images were resampled to 1.6 mm isotropic resolution with a bspline interpolation (axi_rs, cor_rs, sag_rs)
2. An initial template was created from the three images using rigid body registration using the ants.build_template command with 1 iteration (from the antspyx python library)

```
>>> import ants

>>> template_rigid = ants.build_template(image_list=[axi_rs, cor_rs, sag_rs],
iterations=1, kwargs={'type_of_transform':'Rigid'}
```

3. Based on the rigid template we run the following command to build the final template

```
$ antsMultivariateTemplateConstruction2.sh -d 3 -g 0.2 -A 2 -i 4 -k 1 -f [2,1] -
s [1,0]vox -q [200,30] -t BSplineSyn -m MSQ -c 2 -j 3 -r 0 -n 0 -o
MY_Deformable_ -z rigid_template_fname inputs_axi.nii.gz inputs_sag.nii.gz
inputs_cor.nii.gz'
```

which comes down to use the following options

Flag	Description	Comment
-g	gradient step	
-i	Iterations	
-k	Number of modalities	Equal to 1 since we only have one image contrast
-f	Shrink factors	
-s	Smoothing factors	
-q	Max iterations	
-t	Transform type	Found empirically that BsplineSyn worked well

-m	Metric	Used mean squares (MSQ) since we have identical image contrast for all orientations
-c	Computation mode	Allow to run in parallel
-j	CPU cores to use	Run each axis on separate core
-r	Rigid-body reg to template	Not using this
-n	N4BiasFieldCorrection	Not using this since it is applied on the scanner
-o	Output file	
-z	Initial template	File created in the previous step

4. Finally, we run HD-BET for skull stripping on the isotropic T_2w image [25].

Image registration

The main registration task is to warp the FreeSurfer SamSeg masks from the isotropic T_2w image space to the native space of the T_1 map. We chose to do this in multiple steps to optimize registration accuracy.

1. The DICOM image of the TI=91 ms image was co-registered with the T_2w isotropic using an affine transformation. The TI=91 ms was chosen as it had good WM to GM contrast to aid in the registration. We chose the DICOM image instead of the BART reconstructed image since the DICOM image had gradient non-linearity correction applied, similarly to the T_2w image.
2. Next, we calculated the gradient non-linearity on the BART reconstructed data by registering the TI=91 ms DICOM to the equivalent BART reconstructed image. We expected this transformation to be non-linear, but smoothly varying. We achieved this through the following steps:
    a. Apply N4 bias field correction to both images.
    b. Co-register the two images with an affine transform using a pyramid scheme.

```
$ antsRegistration --dimensionality 3 --float 0 --collapse-output-transforms 1
                --output [
{aff_out}_,{aff_out}_Warped.nii.gz,{aff_out}_a_InverseWarped.nii.gz ]
                --interpolation Linear
                --use-histogram-matching 0
                --winsorize-image-intensities [ 0.005,0.995 ]
                --initial-moving-transform [ {aff_ref},{aff_move},1 ]
--transform Rigid[ 0.1 ]
                --metric MI[ {aff_ref},{aff_move},1,32,Regular,0.25 ]
                --convergence [ 500x250x100,1e-6,10 ]
                --shrink-factors 4x2x1
                --smoothing-sigmas 2x1x0vox
--transform Affine[ 0.1 ]
                --metric MI[ {aff_ref},{aff_move},1,32,Regular,0.25 ]
                --convergence [ 500x250x100,1e-6,10 ]
                --shrink-factors 4x2x1
                --smoothing-sigmas 2x1x0vox
```

c. Calculate the non-linear registration with a SyN transform, using the affine from step 2 as the initial affine transform. In this step the field_var is restricted to X to produce a smoothly varying deformation field.

```
$ antsRegistration --dimensionality 3 --float 0 --collapse-output-transforms 1
                    --output [
{syn_out}_,{syn_out}_Warped.nii.gz,{syn_out}_InverseWarped.nii.gz ]
                    --interpolation {interp}
                    --use-histogram-matching 0
                    --winsorize-image-intensities [ 0.005,0.995 ]
                    --initial-moving-transform {syn_init}
          --transform SyN[ 0.1,30,0 ]
          --metric CC[ {syn_ref},{syn_move},1,4 ]
          --convergence [ 750x500x100x70,1e-6,10 ]
          --shrink-factors 8x4x2x1
          --smoothing-sigmas 4x2x1x0vox
```

d. The three registration steps (T_2w iso -> T_1w DICOM, T_1w DICOM -> (affine + SyN) -> T_1w BART recon) are then combined into a single interpolation step and the FreeSurfer SamSeg masks are warped to the T_1 map space using a generic label interpolator.

S3. Phantom temperature for each session at each site

An analysis of the relationship between phantom temperatures and T_1 estimates is presented here, including temperature measurements across sites and sessions.

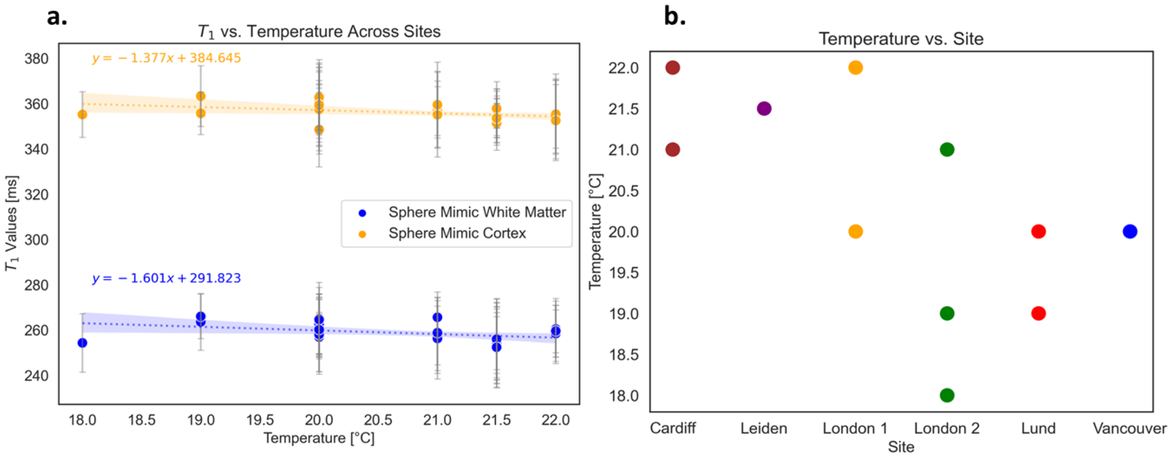

Figure S3. T_1 vs. temperature measured in two spheres of the phantom, with three scans conducted at each site **(a).** The regression lines for both spheres show a minimal slope, suggesting that temperature has little effect on the T_1 estimates. Error bars represent the spatial standard deviation within the ROI for each tube. Panel **b.** displays the temperature variation observed at each site.

Table S2 *: Phantom temperature recorded at each site across three sessions.*

Site	T(°C)Session 1	T(°C)Session 2	T(°C)Session 3
Cardiff	21	22	21
Leiden	22	22	22
London 1	22	22	20
London 2	18	19	21
Lund	19	20	20
Vancouver	20	20	20

S4. Specifics of skewness and FWHM for the T_1 distributions of WM and cerebral cortex at each site

The details of the skewness and Full-Width at Half Maximum (FWHM) measurements for white matter and the cerebral cortex across all sites are presented here.

Table S3 *: Skewness and Full-Width at Half Maximum (FWHM) measurements for white matter and cerebral cortex at each site.*

Site	White Matter		Cerebral Cortex	
	Skewness	FWHM [ms]	Skewness	FWHM [ms]
Cardiff	1.86	72	1.95	62
Leiden	1.77	80	2.04	60
London 1	1.85	72	2.01	60
London 2	2.00	65	2.12	57
Lund	1.83	72	1.92	65
Vancouver	1.89	69	2.00	62

S5. Tukey Honest Significant Difference (HSD) post-hoc test results for pairwise comparisons between brain regions.

For each pair of brain regions, the mean difference in measurements is calculated (averaged across left and right hemispheres) and p-values are adjusted using Tukey's method to account for multiple pairwise comparisons. Significant differences (null hypothesis rejected at $\alpha = 0.05$) are indicated by two asterisks (**) next to the adjusted p-value.

Table S4 *: Tukey's HSD post-hoc test results for pairwise regional comparisons. Adjusted p-values are reported for all comparisons. Significant differences (p < 0.05) are marked with two asterisks (**).*

Group 1	Group 2	p-adj
Caudate	Cerebellum-Cortex	0.30**
Caudate	Cerebellum-White-Matter	< 1e-10
Caudate	Cerebral-Cortex	0.0006
Caudate	Cerebral-White-Matter	< 1e-10
Caudate	Putamen	< 1e-10
Caudate	Thalamus	< 1e-10
Cerebellum-Cortex	Cerebellum-White-Matter	< 1e-10
Cerebellum-Cortex	Cerebral-Cortex	0.39**
Cerebellum-Cortex	Cerebral-White-Matter	< 1e-10
Cerebellum-Cortex	Putamen	< 1e-10
Cerebellum-Cortex	Thalamus	< 1e-10
Cerebellum-White-Matter	Cerebral-Cortex	< 1e-10
Cerebellum-White-Matter	Cerebral-White-Matter	< 1e-10
Cerebellum-White-Matter	Putamen	0.035
Cerebellum-White-Matter	Thalamus	0.38**
Cerebral-Cortex	Cerebral-White-Matter	< 1e-10
Cerebral-Cortex	Putamen	< 1e-10
Cerebral-Cortex	Thalamus	< 1e-10
Cerebral-White-Matter	Putamen	0.0002
Cerebral-White-Matter	Thalamus	< 1e-10
Putamen	Thalamus	0.95**
